# Evidence of Early-Stage Selection on *EPAS1* and *GPR126* Genes in Andean High Altitude Populations

**DOI:** 10.1038/s41598-017-13382-4

**Published:** 2017-10-12

**Authors:** Christina A. Eichstaedt, Luca Pagani, Tiago Antao, Charlotte E. Inchley, Alexia Cardona, Alexander Mörseburg, Florian J. Clemente, Timothy J. Sluckin, Ene Metspalu, Mario Mitt, Reedik Mägi, Georgi Hudjashov, Mait Metspalu, Maru Mormina, Guy S. Jacobs, Toomas Kivisild

**Affiliations:** 1 Thoraxclinic at the University Hospital Heidelberg, Heidelberg, Baden-Württemberg Germany; 2 Institute of Human Genetics, Heidelberg University, Heidelberg, Baden-Württemberg Germany; 3 Department of Archaeology and Anthropology, University of Cambridge, Cambridge, Cambridgeshire UK; 4 Estonian Biocentre, Tartu, Tartumaa Estonia; 5 Division of Biological Sciences, University of Montana, Missoula, Missoula County, Montana USA; 6 MRC Epidemiology Unit, University of Cambridge, Cambridge, Cambridgeshire UK; 7 Institute for Computational Biology, University of Montpellier, Montferrier-sur-Lez, Hérault France; 8 Mathematical Sciences, University of Southampton, Southampton, Hampshire UK; 9 Department of Evolutionary Biology, Institute of Molecular and Cell Biology, University of Tartu, Tartu, Tartumaa Estonia; 10 Estonian Genome Centre, University of Tartu, Tartu, Tartumaa Estonia; 11 Department of Biotechnology, Institute of Molecular and Cell Biology, University of Tartu, Tartu, Tartumaa Estonia; 12 Statistics and Bioinformatics Group, Institute of Fundamental Sciences, Massey University, Palmerston North, Kairanga New Zealand; 13 Department of Applied Sciences, Faculty of Humanities and Social Sciences, University of Winchester, Winchester, Hampshire UK; 14 Complexity Institute, Nanyang Technological University, Singapore, Singapore

## Abstract

The aim of this study is to identify genetic variants that harbour signatures of recent positive selection and may facilitate physiological adaptations to hypobaric hypoxia. To achieve this, we conducted whole genome sequencing and lung function tests in 19 Argentinean highlanders (>3500 m) comparing them to 16 Native American lowlanders. We developed a new statistical procedure using a combination of population branch statistics (PBS) and number of segregating sites by length (nSL) to detect beneficial alleles that arose since the settlement of the Andes and are currently present in 15–50% of the population. We identified two missense variants as significant targets of selection. One of these variants, located within the *GPR126* gene, has been previously associated with the forced expiratory volume/forced vital capacity ratio. The other novel missense variant mapped to the *EPAS1* gene encoding the hypoxia inducible factor 2α. *EPAS1* is known to be the major selection candidate gene in Tibetans. The derived allele of *GPR126* is associated with lung function in our sample of highlanders (p < 0.05). These variants may contribute to the physiological adaptations to hypobaric hypoxia, possibly by altering lung function. The new statistical approach might be a useful tool to detect selected variants in population studies.

## Introduction

High altitude represents an extreme environment characterised by low concentrations of atmospheric oxygen (hypoxia), arid climate, high solar radiation and other environmental stressors. Populations have resided at high elevations in Ethiopia, the Himalayas and the Andes for several millennia^1^. Each of these populations, faced with ongoing environmental pressure, has developed their unique array of physiological adaptations. In Andeans, these include an enlarged chest, increased lung capacities^2^, only slightly increased ventilation rate and an elevated haematocrit^3^.

In this study we focus on the Colla population living in the Northwest Argentinean highlands. Collas also inhabit Southern Bolivia and Northern Chile and are considered to be related to other Andean groups such as Quechua, Aymara and Atacameño^4^. These groups could trace back to the beginning of human settlement in the Andes, which archaeological evidence places between 12,000 and 9,000 years before present^5–7^.

The genetic component of high altitude adaptation in Andeans has been the subject of a number of recent studies^8–10^. Using genotype data genes such as *VEGFB, PRKAA1*, *NOS2A* and *FAM213A* have been suggested to be under selection in Andeans^8–10^. These genes play a role in a vast array of processes such as cardiac function, oxygen sensing, vasodilation and oxidative stress reduction. In Tibetans, a particular gene involved in the response to hypoxia, the hypoxia inducible factor 2α (HIF-2α) or *EPAS1*, has been repeatedly shown to have a signature of selection^11–14^ and to be associated with the almost absent increase of haemoglobin concentration in native Himalayan populations^11,14^. A gene involved in the oxygen sensitive expression of HIF itself, *EGLN1*, was selected in both Tibetans and Andeans^12^. A first subset of Andean whole genome sequences was published comparing 10 healthy Peruvian highlanders with highlanders suffering from chronic mountain sickness^15^. The authors detected variants in an erythropoiesis regulator gene *SENP1*, and an oncogene, *ANP32D* in association with cross-population tests of selection and a higher transcriptional response to hypoxia in individuals with chronic mountain sickness relative to those without.

Compared to other high altitude populations, such as Tibetans, Andeans have been living for less time at elevations above 3500 m. Given that there has been shorter duration for selection to act, rather than being close to fixation, it is likely that proportionally more advantageous gene variants exist at intermediate frequencies. To this end, we complement the scans for hard sweep variants with scans for signatures of incomplete selective processes. These tests are applied on high coverage whole genome sequence data for healthy Andean highlanders from Northwest Argentina together with sequence data from Native American lowlanders and pinpoint to variants advantageous in the adaptation to high altitude.

## Results

The sequencing of the whole genome of 19 Collas living above 3500 m resulted in an average genome-wide call rate of 97.4% (min. 97.1%). Coverage of 30x was reached for 95% of the genome with a minimum of 89% in one individual, while the minimum exonic coverage was 93%. On average 280 Gb of sequence data could be mapped for each genome with a minimum of 205 Gb in a single individual. Around 3.3 million SNPs could be identified in each highlander genome. Of these 1.7% were novel SNPs. Within the whole exome only 20,900 SNPs were identified with 2% representing newly discovered SNPs (on average for each individual per genome compared to a reference genome). About 10,200 synonymous SNPs had no effect on the amino acid sequence while 9,350 were non-synonymous. These were further classified as: mainly missense mutations (9,250, average value of all individuals), about 75 nonsense, 10 nonstop, 18 misstart and 70 disruptive mutations.

**Figure 1:**
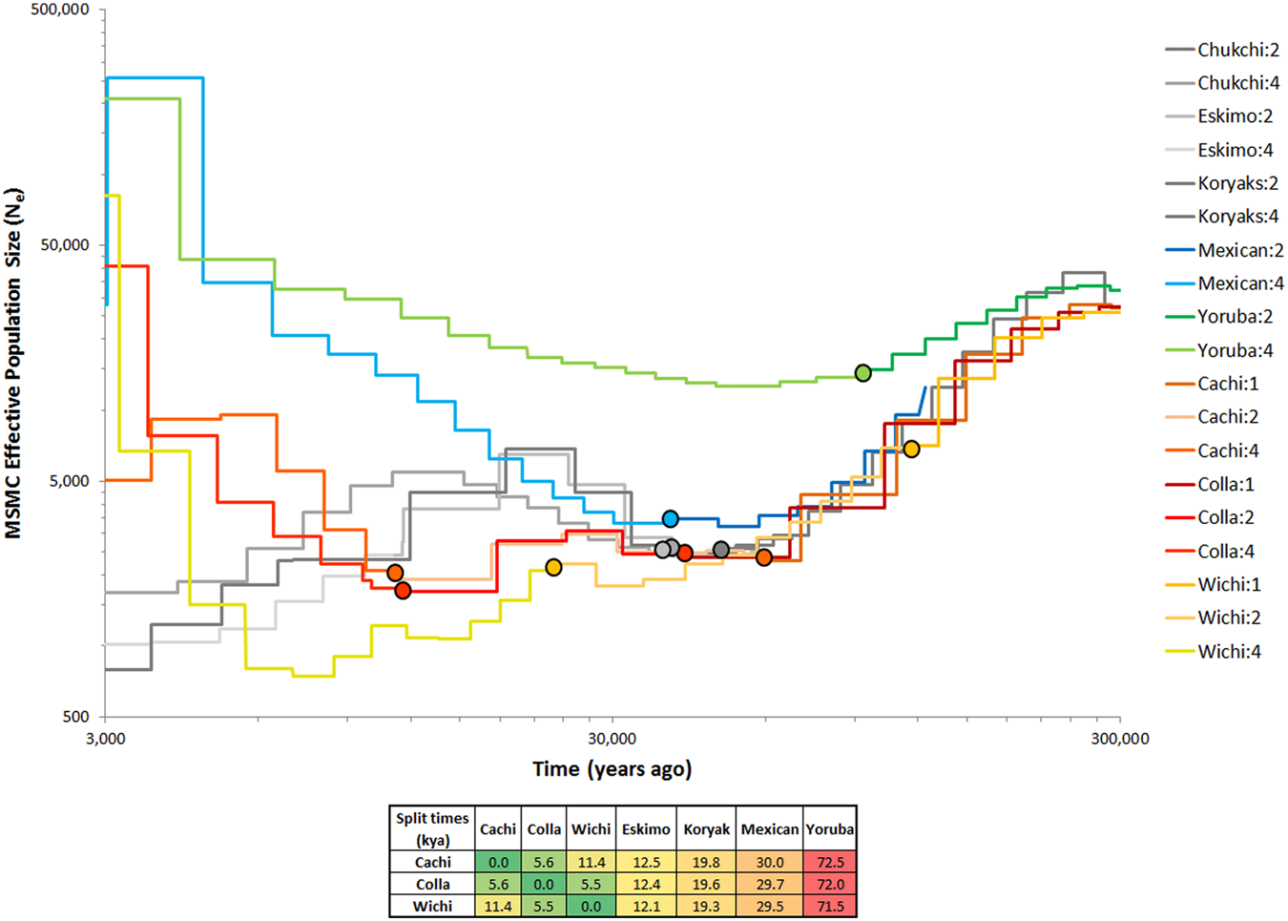
MSMC plot, effective population sizes and split times of Collas, Native American lowlanders, Siberians and Africans. Effective population size (N_e_) and split time estimates are based on 1, 2 or 4 genomes for the Native Americans and on 2 and 4 genomes for the other populations. Coloured dots show where the MSMC curves based on 1, 2 or 4 genomes were joined together to provide a comprehensive representation of the changes in N_e_ over time for each of the analysed population. Around 100,000 years the out of Africa exit starts to reduce population size in non-Africans. Yoruba show a limited decline as they remain in Africa. N_e_ of Collas rises up to 30,000 units 3,000 years ago. Andean highlanders: Collas; Calchaquíes (Cachi): intermediate altitude population in Argentina (2300 m); American lowlanders: Mexican, Wichí; Siberians: Eskimo, Koryaks, Chukchi.

To investigate population structure, effective population size and split times of highlanders and control populations in the last 300,000 years we conducted multiple sequentially Markovian coalescent analyses (MSMC, Fig. 1). All non-African populations showed a similar pattern 30,000–300,000 years ago dominated by the out of Africa exit. Split dates of non-African populations were around 12,000–30,000 years. Population divergence was consistent with a single Eurasian origin for all analysed populations. The neighbouring populations Collas and Calchaquíes only separated in the last 5,000 years (Fig. 1). Calchaquíes showed high genetic similarity but also population specific differences to the Collas^2^. The divergence time of Collas and lowland Wichí was estimated to be 5,000 years possibly indicating the onset of permanent high altitude settlement (Fig. 1). The genetic effective population size of Collas declined to 1500 around 9000 years ago and then rose up to 30,000 at 3,000 years ago. The total census size in 2005 of the Collas was estimated to be around 70,500 including Collas residing at lower altitudes^16^.

### Signals for positive selection in Collas

We first searched for alleles with high-frequency differences between Collas and other populations that could be driving late-stage selective sweeps. We retrieved all SNPs located in top 1% Tajima’s D^17^ and number of segregating sites by length (nSL)^18^ 200 kb windows, and then filtered these for variants in the top 1% of population branch statistic (PBS)^14^ scores. PBS is a statistic that detects variants that have changed substantially in frequency in a target population, using a related population and an outgroup to determine which population the allele frequency change occurred in. The nSL test is a haplotype based statistic identifying haplotypes that are at high frequency but haven’t yet been broken up by recombination, suggesting that the increase in frequency was recent. Both statistics seek to identify a recent change in the frequency of an allele or haplotype, which can reflect the impact of selection in a genomic region. PBS was calculated for 19 Collas in comparison to 19 Siberians^19,20^ and 16 lowlanders from Central and South America^2^. The lowland group represented a conservative grouping since it included Calchaquíes, who live at an altitude of 2300 m in Argentina and bear overall genomic similarities to the nearby Collas^2^. Hence, any genomic signatures shared by Calchaquíes and Collas would be missed by our approach. These filters yielded 72 SNPs. After excluding SNPs with frequency >10% in the 1000 Genomes database, those without gene annotation, and with combined annotation dependent depletion (CADD) scores ≤5 only 9 SNPs remained (Table 1).

**Table 1:** Top 1% PBS alleles located within top 1% nSL windows.

Chr: position	Identifier	Allele frequency Collas	Allele frequency Lowlanders	Allele frequency Siberians	1000 Genomes DAF	Impact	Associated gene	PBS p-value
11:67209515	rs111451405	74%	44%	3%	4%	syn	*CORO1B*	0.001
11:67072382	rs61731786	71%	44%	3%	7%	syn	*SSH3*	0.002
11:67076748	rs12417770	71%	44%	3%	7%	intronic	*SSH3*	0.002
11:67159257	rs17880138	71%	44%	3%	7%	promoter	*RAD9A*	0.002
11:67165015	rs2066494	71%	44%	3%	6%	syn	*RAD9A*	0.002
11:67057599	rs1574103	71%	47%	3%	4%	syn	*ANKRD13D*	0.005
6:159062992	rs882735	63%	38%	0%	2%	intronic	*DYNLT1*	0.006
20:2840929	rs2297048	58%	25%	18%	4%	syn	*VPS16*	0.008
20:2844911	rs2274671	58%	25%	18%	5%	syn	*PTPRA; VPS16*	0.008

Chr = chromosome, DAF = derived allele frequency, syn = synonymous; CADD score for all SNPs > 5.

All of these SNPs were highlighted by the haplotype test nSL and none by Tajima’s D. Notably, six of the nine remaining SNPs were found in the same haplotype context located on chromosome 11 at 67 Mb. Interestingly, this region is 3 Mb upstream of the highest ranking region highlighted previously by the integrated haplotype score (iHS) test in Collas^9^. The SNP with the highest CADD score of 19.11 was part of the *ANKRD13D* gene which codes for an ubiquitin binding protein. All hits were in non-coding regions and also showed substantial allele frequency in the lowland population (Table 1).

**Figure 2:**
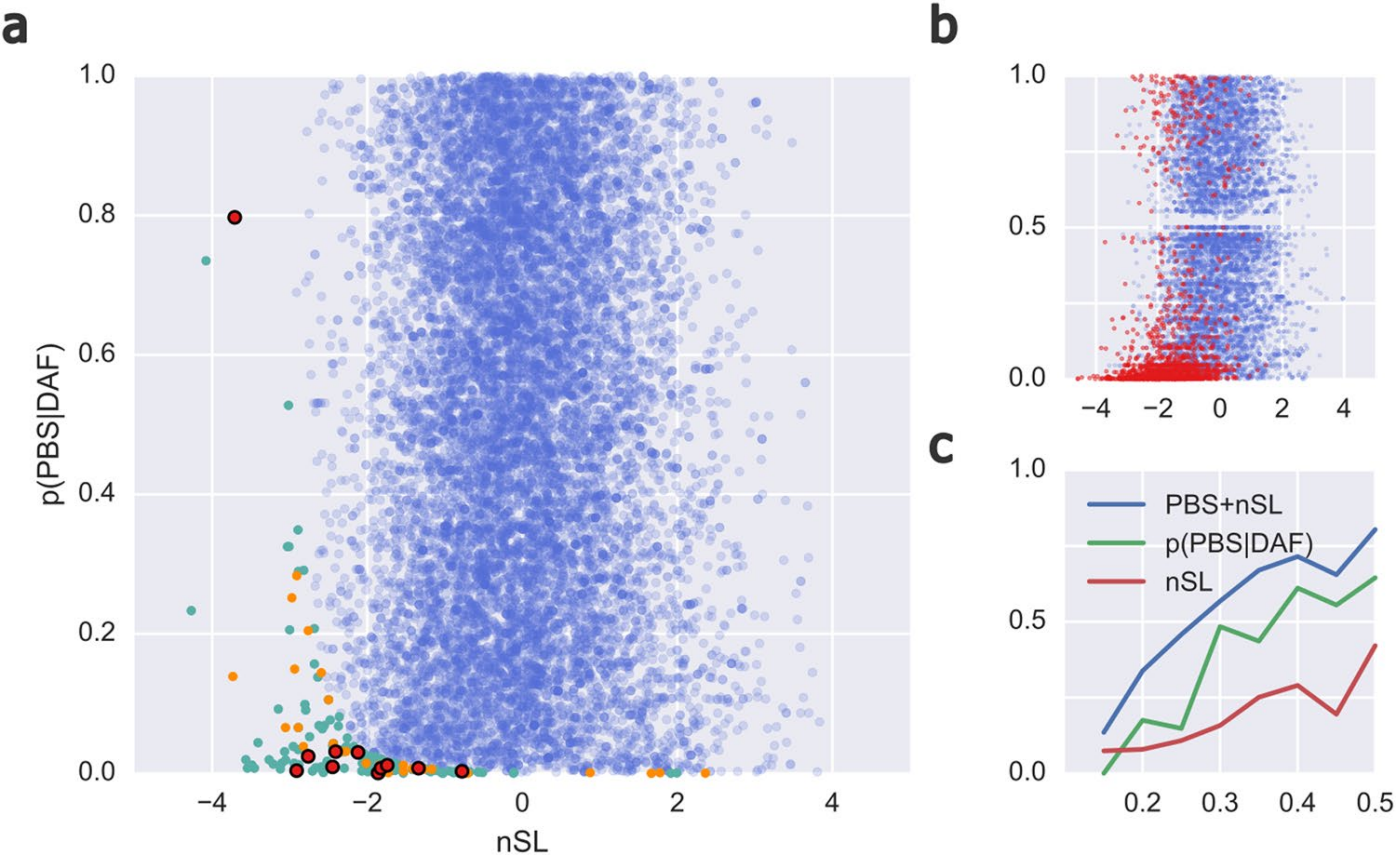
Distribution of nSL + PBS and power calculations. (**a**) Observed joint distribution of nSL and p(PBS|DAF) of all exonic SNPs (blue), with top-1% outliers coloured teal (not missense), orange (missense) or red with black border (missense; the 11 highly damaging variants). (**b**) Simulated joint distribution of nSL and p(PBS|DAF), with X- and Y-axes as in (a). Results from neutral scenarios are shown in blue, with those from selected scenarios (s = 0.02) in red. Note the clustering of selected SNPs in the low-p(PBS|DAF), low-nSL regime, corresponding to that detected by our PBS + nSL selection statistic; also note the especial clustering of most damaging missense variants in (a) in this region of the plot. (**c**) Power from the simulations of the three statistics to detect selection, with selected variants with a p-value ≤ 0.0062 classified as successful detections. X- and Y-axes are derived allele frequency and power, respectively.

Given the short time-frame of human occupation in the Andes^21^, we hypothesize that ongoing selective sweeps acting on intermediate frequency selected variants may contribute a substantial fraction of adaptive genetic changes among the Collas. However, detecting a selective sweep in its early stages poses particular challenges. When selection has not yet driven an allele to high frequency, the impact on linked genetic variation is also low. Consequently, most statistics designed to detect positive selection only have high power towards the final stages of a selective sweep^22–24^. Using simulations to inform our targeted approach, we chose to combine several aspects of the population genetic data and genome annotation information. To search for these signals, we focused on derived allele frequency (DAF) ≥ 15% and ≤50% to identify alleles at the beginning of selection which are overlooked in the traditional positive selection tests. To this end, we obtained population branch statistic (PBS) and number of segregating sites by length (nSL) scores for exonic variants with a DAF ≥ 15% and ≤50% in Collas, corresponding to 17147 SNPs of which 2831 (16.5%) were classified as missense variants in dbSNP build 141. Calculating nSL + PBS scores as described in the methods (Fig. 2a) yielded a similar score distribution as retrieved from simulations (Fig. 2b); these simulations further suggested that combining the two methods results in a greater power to identify intermediate frequency sweeps than each method on its own (Fig. 2c). This approach yielded 42 missense hits (Supplementary Table S1), as compared to the 28 expected missense SNPs (significant enrichment, corrected Chi Square test, one-sided p = 0.0023). PBS, nSL, and p(PBS|DAF_Colla_) statistics used alone yielded 25, 31 and 31 hits respectively (one-sided p-value not significant). Assuming missense variants are more likely to have an adaptive phenotypic effect, the data supports simulations in suggesting that combining p(PBS|DAF_Colla_) and nSL scores increases power to detect early-stage selection. Based on the degree of enrichment, we expect approximately 14/42 top missense hits to be caused by non-neutral processes.

We found that 23/42 (54.8%) missense hits had evidence of a deleterious effect (as indicated by a deleterious SIFT or PolyPhen2 categorisation), compared to 960/2733 (35.1%) over all scored missense variants. This enrichment (corrected Chi-Square, one-sided p-value = 0.0066) suggests that PBS + nSL outliers are likely to be damaging. Given that we would expect 42 * 0.344 ≈ 14.7 damaging hits in 42 random missense variants, finding 23 suggests an excess 8–9 damaging missense variants that may be non-neutral.

The enrichment of selection signatures among deleterious missense variants with outlier PBS + nSL scores prompted us to define a set of variants with especially high-probability of being damaging. We supplemented SIFT and PolyPhen2 scores with CADD scores for our 42 missense hits and defined a high-probability damaging variants as (SIFT: “damaging”, PolyPhen2: “possibly damaging” or “probably damaging”, and CADD Phred >5). This identified 11 SNPs (Table 2).

**Table 2:** Top PBS + nSL missense hits based on intermediate derived allele frequencies between 15–50%.

Chr: Position	Identifier^a^	Ref	Alt	Allele frequency Collas	Allele frequency Lowlanders	Allele frequency Siberians	1000 Genomes DAF	Gene	Amino acid change	PBS + nSL score
8:24349417	rs3736281	T	C	47%	6%	11%	2.8%	*ADAM7*	I453T	0.000
6:142688969	rs17280293	A	G	50%	3%	8%	2.8%	*GPR126*	S123G	0.000
21:33689199	rs762225	G	C	34%	3%	5%	1.4%	*URB1*	P2071R	0.001
1:40766943	rs140041506	G	T	37%	6%	5%	0.7%	*COL9A2*	P661T	0.001
14:58958923	rs3783697	A	G	29%	6%	32%	2.4%	*KIAA0586*	D1263G	0.001
14:33165230	rs543290190	T	C	16%	0%	0%	0.1%^b^	*AKAP6*	W972R	0.002
3:77629200	rs188582283	C	T	24%	0%	0%	0.9%	*ROBO2*	R811W	0.002
2:46588031	rs570553380	A	G	32%	6%	0%	0.2%^c^	*EPAS1*	H194R	0.004
6:76631823	rs76604824	C	T	24%	3%	3%	4.6%	*IMPG1*	D793N	0.004
22:30888494	rs17738527	C	T	50%	16%	0%	14.4%	*SEC. 14L4*	E211K	0.005
16:3708170	rs2791	C	T	24%	0%	0%	1.7%	*TRAP1*	R692H	0.006

^a^All variants were classed as possibly/probably damaging in PolyPhen 2 and damaging in SIFT and had a CADD score > 17, for values see Supplementary Table S1

^b^Only found in 4 heterozygote Peruvians from Lima out of 85 individuals. ^c^Only found in 12 heterozygote Peruvians from Lima out of 85 individuals; lowland allele frequency strongly influenced by 20% allele frequency in Calchaquíes (n = 2). Chr = chromosome, Ref = reference allele, Alt = alternative allele, DAF = derived allele frequency.

**Figure 3:**
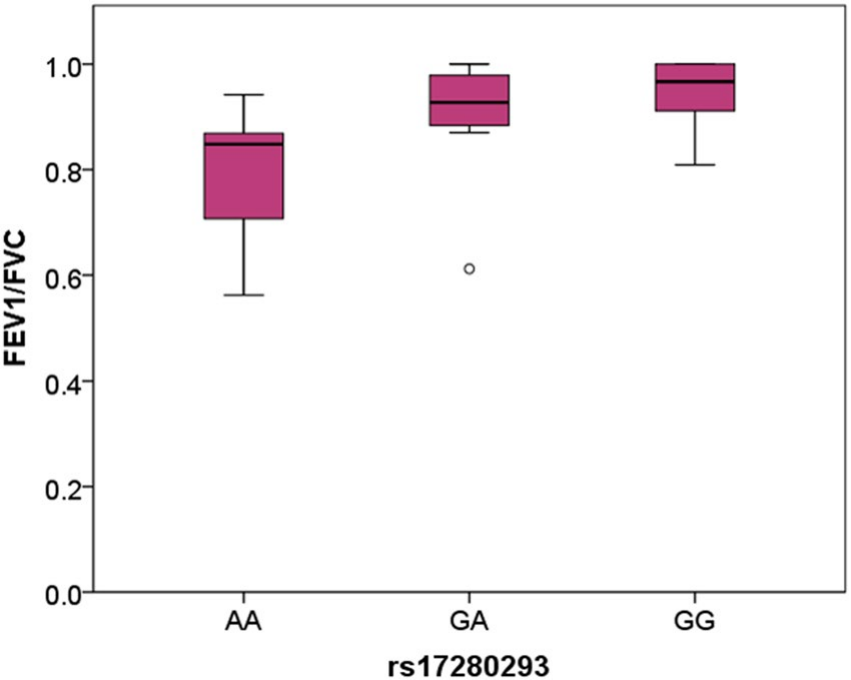
FEV1/FVC in Collas sorted by derived allele frequency. The ratio increases with number of derived alleles indicating an improvement of lung function in Collas. One-tailed p-value = 0.029, r^2^ = 0.195; FEV1: forced expiratory volume in the first second, FVC: forced vital capacity.

Of these 11 SNPs, two (rs570553380 and rs543290190) are not found in 1000 Genome Phase 1 data and are polymorphic only in Peruvians from Lima in the extended 1000 Genome Phase 3 data set; this was only true for one other variant in our list of 42 missense hits. Tellingly, one of these two variants (rs570553380) is located in the *EPAS1* gene, which is a major oxygen sensor and has been identified multiple times as subject to high altitude selection in Tibetans^11–14^. The other SNP (rs543290190) is located in *AKAP6*, which is involved in cardiac myocyte reaction to stresses such as hypoxia, hypertrophic remodelling of the heart^25^ and stabilises the hypoxia master regulator HIF-1α during hypoxia while enhancing its degradation at normoxia^26^. Furthermore, of the 11 shortlisted SNPs, rs17280293, located in *GPR126*, is the variant with the second most extreme nSL + PBS score. During the course of our data analysis, a study of a European cohort was published identifying a strong association between lung function and SNP rs148274477 (largest effect size and most extreme p-value reported in that study, p = 9.58e^−26^)^27^. In that cohort, the *GPR126* SNP rs17280293 that we identify here is mentioned as being in strong LD with rs148274477 (r^2^ = 0.85). While the same level of LD is observed in our Colla population (r^2^ = 0.848) the intriguing possibility exists that it, rather than rs148274477, is driving a strong lung function association in a population unrelated to the Colla. The derived alleles at both SNPs in *GPR126* rs148274477 and rs17280293 are associated with increased forced expiratory volume in the first second/forced vital capacity (FEV1/FVC). As we have FEV1/FVC measurements for our 19 Colla individuals^2^, we were able to speculatively test for a similar association in our small Colla sample. We could identify a positive association (Fig. 3; one-tailed p = 0.029) with each additional derived allele at rs17280293 associated with an increase in FEV1/FVC of 0.075 (standard error = 0.037). While this association does not persist when phenotypic and genetic data from Wichí (a lowland population) and Calchaquíes (an intermediate altitude population) are incorporated into the linear regression (one-tailed p = 0.158, data not shown) there could be several confounding factors such as population structure or an effect on FEV1/FVC associated with the altitude at which the lung function measurements were taken.

Based on a wide range of evidence - low worldwide frequency DAF (1000 Genome) and local population differentiation (PBS), relatively extended derived-allele haplotype homozygosity (nSL) and evidence for disruption of the protein (SIFT, PolyPhen2, CADD) – as well as evidence based on prior selection signals and gene function, we consider the two missense variants in *EPAS1* and *GPR126* as high-probability selection candidates.

## Discussion

In this study, we have detected missense variants as novel candidates for local positive selection in the genomes of Andean highlanders with possible functional consequences. By combining different selection statistics, along with genome annotation information, we were able to identify intermediate frequency variants that may contribute to high altitude adaptation in two very prominent candidate genes, *GPR126* and *EPAS1*. The first gene has been previously associated with lung function measurements; the second, with the main adaptive phenotype in Tibetans. In contrast, no functional variant could be identified in Collas by scans of late-stage selective sweeps.

Our first main finding is the second strongest hit in the intermediate frequency analysis originated from a missense SNP (rs17280293) in *GPR126* (or *ADGRG6, Adhesion G Protein-Coupled Receptor G6*) found at 2.8% in 1000 Genomes Project data. This mutation causes an amino acid change (p.S123G) classed as damaging by SIFT and possibly damaging by PolyPhen2 within the seven transmembrane domain of the protein. Several previous studies have shown a strong association of this gene with lung function^27,28^. Furthermore, this SNP has been identified as in strong linkage disequilibrium with a variant (rs148274477) associated with FEV1/FVC. We found rs17280293 to be associated with FEV1/FVC in our own phenotype data, suggesting that Andeans with a derived allele may have better equipped lungs for life at high altitude. This is the first time a phenotype in Andeans could be correlated with genomic data. Taken together, the extreme PBS + nSL score, evidence of damaging effect, and association with lung function situate rs17280293 as an interesting selection candidate.

Our second main finding highlighted a missense SNP in the hypoxia inducible factor 2 α (HIF-2α, *EPAS1*) at intermediate frequencies in the Collas. While the HIF-1α is activated during short-term hypoxia, HIF-2α is rather expressed as a chronic hypoxia response^29^. Both transcription factors regulate a plethora of genes involved in energy metabolism and differential oxygen use during hypoxic conditions. *EPAS1* has already been repeatedly shown to have a signature of selection in Tibetan populations^11–14,30^, but this is the first study to indicate selection also in Andeans. Since this gene was highlighted with a test investigating variants at intermediate levels it may indicate that a potentially advantageous allele of *EPAS1* is on its way to higher frequencies also in Andeans but has not yet reached levels equivalent to Tibetans. Interestingly, the selected haplotype in Sherpas and Tibetans was suggested to be an introgression from the Denisovan genome^31^ and its frequency increased with altitude of populations in the Himalayas^32^. The Tibetan genotype has been associated with an inhibition of haemoglobin concentrations rise at high altitude^11,14^. However, the specific functional mechanism has not yet been identified in Tibetans. This might be due to the fact that all highlighted SNPs so far fall within non-coding regions of the gene^33^. In contrast, the highlighted variant in Andeans (rs570553380) encodes a missense mutation predicted to have a damaging effect on protein function. Knock-out studies in mice underlined the essential role of HIF-2α for normal erythropoiesis. Complete knock-out leads to a drastically reduced haematocrit^34^. Heterozygous knock-out mice had a similar haematocrit compared to wild type mice but showed a resistance to hypoxia induced pulmonary hypertension and thus no subsequent right ventricular hypertrophy^35^. This functional advantage under hypoxic conditions suggests a protective role of at least some loss of function mutations in *EPAS1* for life at high altitude. In contrast, human patients with gain of function mutations were characterised by pulmonary hypertension and erythrocytosis showing a continuous activation of the HIF response^36^. Hypoxia induced pulmonary hypertension is a universal physiological response to lowlanders going to high altitude^37^ and is constantly present in Andeans^38^. While in healthy highlanders pressures are only moderately increased^39^ they are exaggerated in individuals suffering from chronic mountain sickness, which is more common in the Andean population than in other high altitude dwellers^40^. Pulmonary hypertension leads to a remodelling of the pulmonary vasculature, inducing changes which may result in right heart failure. Thus, a protective allele in *EPAS1* might not only reduce the red blood cell count as seen in Tibetans but also protect from extensive pulmonary hypertension in the Andean population.

We based our results on 19 whole genome sequences from the Argentinean Collas. The results of this study should be confirmed in a larger sample possibly including further Andean high altitude populations. Additionally, further work to clarify the effect of the *EPAS1* variant, for example by assessing any association with haematocrit in these groups, would be valuable.

In conclusion, we introduce a new approach for detecting early-stage selective sweeps which enables us to identify moderate frequency (0.15–0.50) derived variants in Andean high altitude populations as potential targets of recent selection. Among exonic variants with functional annotation we identify two missense variants in the *GPR126* and *EPAS1* genes. *GPR126* is known to be involved in lung function and the selected *EPAS1* variant might have a potential protective effect on hypoxia induced erythrocytosis and pulmonary hypertension. In case of the *EPAS1* gene, our results are the first to point to a plausible target of selection at variant level. Considering this variant is restricted to South America these results provide strong evidence of convergent evolution in Andeans and Tibetans living with the same environmental pressures on opposite sides of the world. The approach of detecting early-stage selective sweeps has the potential to be applied to other cases of recent local selection.

## Methods

### Subjects and ethical approval

All participants provided their written informed consent. The study was approved by the by the Ethics Committee at the University of East Anglia and the Ministry of Health of the Province of Salta (Ministerio de Salud Pública, Salta, Argentina). Ethical approval for the whole genome sequencing of the samples was obtained from University of Cambridge Human Biology Research Ethics Committee (HBREC.2011.01). All methods were performed in accordance with the relevant guidelines and regulations. High altitude samples were collected from an Andean population, the Colla, living in Northern Argentina at an altitude >3500 m^9^. Native American lowland control samples for selection analyses originated from 5 Calchaquí and 4 Wichí from neighbouring regions in Argentina^2^, plus publicly available sequences from the Complete Genomics database including 5 Mexican (NA19648-200-37-ASM, NA19669-200-37-ASM, NA19735-200-37-ASM, NA19649-200-37-ASM, NA19670-200-37-ASM) and 2 Puerto Rican (HG00732-200-37-ASM, GS000015711-ASM) samples^20^. Additional North-East Siberian samples served as controls for threesided population statistics including 2 Chukchi, 4 Eskimo and 13 Koryak individuals^19,20^. Two additional Chukchi individuals were added in the demographic analyses.

### Whole genome sequences

Whole genome sequences were obtained with the Complete Genomics platform and quality filtered as described elsewhere^20^. The newly sequenced Colla genomes were deposited in the European Nucleotide Archive: PRJEB14828. Data are also freely available through the Estonian Biocentre website. All genomes were annotated against the Ensembl GRCh37 database and compared to dbSNP Human Build 141 and Phase 1 of the 1000 Genomes Project dataset^41^ as described previously^20^. For 1000 Genomes variant frequency Phase 3 data^42^ was used. We focused on variants annotated as “exonic” in Ensembl occurring in Hardy-Weinberg equilibrium in our sample resulting in 402,227 autosomal variants. Variants with a combined annotation dependent depletion (CADD) score >5 were investigated further. The CADD score was calculated to estimate impact of synonymous and non-synonymous variable alike^43^. Missense variants were assessed with the *in silico* prediction programs SIFT^44^ and Polyphen2^45^.

### Tests of positive selection

Two statistics were calculated for each SNP – the population branch statistic (PBS)^14^ and the number of segregating sites by length (nSL)^18^. The PBS score was based on pairwise *F*_ST_ values for the three populations using a weighted analysis of variance^46^ with the program GENEPOP’007^47^. PBS was estimated as in Yi and colleagues^14^. The nSL scores were calculated only on the Colla sample.

Two statistics were window based. We calculated Tajima’s D on the Colla sample using non-overlapping 200 kb windows^17^. A window-based nSL score was also calculated as the average proportion of common (minor allele frequency < 95%) SNPs with |nSL| > 2.0 in a 200 kb window.

In scans for late-stage hard sweeps we first identified the 200 kb windows that showed genome-wide the lowest 1% of Tajima’s D or nSL scores. The top 1% PBS scoring variants in these windows were further filtered for SNPs with less than 10% frequency in the 1000 Genomes Phase 3 data. We only retained those with a CADD score higher than 5.

### Intermediate frequency based selection

As the timescale of human occupation of the Andes is relatively short^21^, we supplemented our selection scans with an attempt to detect positive selection acting on intermediate frequency variants. To this end, we first chose to focus on SNP-by-SNP selection statistics, considering each SNP a potential target of selection. Although this vastly increases the number of tests we performed, early-stage selective sweeps have not yet had a substantial impact on variation far from the selected locus such that they may be difficult to detect using average statistic values or the number of outliers over large genomic windows. We first calculated the two statistics PBS and nSL for each SNP in the Colla sample. For nSL approximate p-values were generated, based on nSL scores that were standardised in derived allele frequency (DAF) bins assuming unstandardised SL follows a roughly normal distribution in frequency bins. PBS values were calculated using lowland Americans as the comparative closely related control population and Siberian populations as outgroup. These were then converted into empirical p-values given DAF in Collas based on 4% DAF bins (e.g. if a PBS value for the 15% ≤ DAF_Colla_ < 19% bin is the 990^th^ largest of 1000 values, its p(PBS|DAF_Colla_) would be 0.01). nSL has been shown to have power to detect early-stage selective sweeps^18^, while PBS has been an effective test-statistic for detecting local selection. Simulations suggest that combining these two statistics using Fisher’s method provides a composite statistic with high power to detect early-stage sweeps (see Fig. 2).

To reduce the number of hypothesis tests and focus on variants for which annotation information exists, we cut our Fisher-combined PBS + nSL score data to SNPs in the exome (Ensemble Biomart B37Rel75), and only considered SNPs with DAF in the Colla of 15% ≤ DAF ≤ 50% to focus on early-stage selective sweeps. We considered the set of top 1% most extreme statistic value SNPs as enriched for SNPs subject to or impacted by selection. We filtered this set for missense variants and obtained CADD, SIFT and Polyphen2 scores for each SNP.

### Multiple sequentially Markovian coalescent analyses

Effective population sizes (N_e_) and population split times between Collas and other Native Americans were estimated using multiple sequentially Markovian coalescent model (MSMC)^48^ on up to four phased genomes per population, as described elsewhere^20^. To provide a comprehensive picture of the changes in effective population size over time of each of the analysed populations, the MSMC curves obtained using 1, 2 or 4 genomes were joined together at points of their overlap. The joining was performed to maximise the MSMC accuracy within a given time interval, as suggested by Schiffels and Durbin^48^ and implemented by Clemente, Cardona and colleagues^49^.

### Simulations

Simulations were performed using the forward-time population genetic simulator SLiM^50^. Briefly, we generated a null distribution of nSL, p(PBS|DAF) and the combined nSL + PBS statistic by generating 250 neutral simulations of the Koryak, Wichí and Colla populations, taking population sizes and population split times from our MSMC analysis. Here, the Colla and Wichí split 5478 years before present (ybp), and the Koryak and ancestral Wichí/Colla population 19451 ybp. These split times are inferred based on cross-coalescence, and no migration is included in the model. We assumed a generation time of 25 years, and started the model at 1 million years ago (with a short, 500-generation burn-in). In each simulation, we generated 1 MB of genetic data, using a mutation rate of 1.7e^−8^/generation and human-realistic variable recombination rate by randomly sampling from non-centromeric regions of the chromosome 2 of the HapMap combined genetic map^51^. At the end of each simulation we retrieved samples according to our study (38 Colla chromosomes, 32 Wichí and 38 Koryak), and then randomly chose 50 mutations with DAF 0.15–0.75 in the Colla sample from the central region (450 kb-550 kb) of the data. We calculated SL and PBS for each of these mutations.

To approximate the distribution of these statistics under selection, we performed 160 simulations following the neutral model until the time of the Colla/Wichí split. At this point, for 20 times in each replicate, we randomly chose a variant in the central region (450 kb-550 kb) and applied positive selection (heterozygote s = 0.01, homozygote derived s = 0.02) for the next 5478 years in the Colla population. We restarted any of the 20 ‘sub-replicate’ routines if the selected allele did not have 0.15 ≤ DAF ≤ 0.75 at sampling time in the Colla sample. On successful completion of a sub-replicate, SL and PBS were calculated for the selected variant.

For both neutral and selected results, SL was normalised to nSL and PBS to p(PBS|DAF) using 5% frequency bins and the neutral distribution of SL and PBS. The nSL score was converted to an approximate p-value by assuming a standard normal distribution of SL in frequency bins, and this was combined with p(PBS|DAF) using Fisher’s method. Each frequency bin included at least 440 neutral results and 100 selected results. Power was estimated using the Fisher-combined p-values with a p-value cut off of 0.0064 (corresponding to the top-1% combined p-value in our genomic data, with weak correlations between nSL and p(PBS|DAF) driving this from the expected value of 0.01).

### Genotype-phenotype assessment

Lung function measurements of forced vital capacity (FVC) and forced expiratory volume 1 second (FEV1) for Collas were reported previously^2^. A ratio of FEV1/FVC was calculated and linear regression was used to assess effects of the derived allele with a one-sided p-value.

## Electronic supplementary material

 Supplementary Information
